# Editorial: Reviews in language sciences

**DOI:** 10.3389/fpsyg.2024.1444503

**Published:** 2024-06-20

**Authors:** Antonio Benítez-Burraco, Antonio Bova, Thomas L. Spalding

**Affiliations:** ^1^Department of Spanish, Linguistics, and Theory of Literature, Faculty of Philology, University of Seville, Seville, Spain; ^2^Department of Psychology, Faculty of Psychology, Università Cattolica del Sacro Cuore, Milan, Italy; ^3^Department of Psychology, Faculty of Arts, University of Alberta, Edmonton, AB, Canada

**Keywords:** language sciences, reviews, theoretical advances, methodological innovations, multidisciplinarity

During the last decades, language sciences have experienced a notable development that has resulted in a deeper understanding of what language is and how it is put into use for fulfilling different functions. Accordingly, we have gained a clearer view of language within the infrastructure of human cognition, including how it is processed by the brain, how it is impaired in people with pathological conditions, how it is acquired by the child, or how it evolved in our species, to name a few. Likewise, we have also made substantial progress in understanding the principles that govern human interaction through language, as observed in daily conversations, but also in many other speech events. Additionally, we now have comprehensive descriptions of thousands of language varieties across the world, from languages to sociolects to registers to styles, as well as detailed characterizations of the physical and sociocultural factors that contribute to regulating such linguistic diversity. Overall, this has notably improved our comprehension of the causes of linguistic diversity, and ultimately, the position of language(s) within human behavior. Methodologically, we now use more sophisticated tools and procedures for analyzing language at all levels, which has resulted in richer data about language facts, and ultimately, in more robust theories about language. Finally, we have also made a significant effort at translating all these discoveries to society, which has crystallized in e.g., better speech therapies aimed to help people with language disorders or more accurate language policies intended to regulate language use in complex, multilingual societies.

All this progress has transformed language sciences into a dynamic and exciting field of research, but at the same time has made research in language sciences very demanding. Researchers in language sciences face several crucial challenges. First, it is not just that, as noted, the amount of data and evidence about language facts has increased exponentially. At present, a proper study of language is not possible without considering the data and evidence provided also by allied disciplines, like archaeology, history, ethology, genetics, or neuroscience, to name a few. In other words, language sciences have become increasingly multidisciplinary in nature. At the same time, and in part as a consequence of such multidisciplinarity, research in language sciences has become more and more demanding from a methodological perspective, resulting in a true technification in many fields. Consider, for instance, the sophisticated facilities used to examine how the brain processes language, the molecular techniques employed to determine the causes of language disorders with a genetic origin, or the Bayesian methods used to create language phylogenies. This methodological challenge is expected to increase during the next decades. Third, given the impressive amount of assorted data about language which is currently available, the time may have come to improve as well our hypotheses about the nature of language, which have been traditionally based on linguistic data (and theory) only. Theorizing better about language will be an additional challenge for the next decades too. Finally, as also noted, researchers in language sciences are more and more concerned about the necessity of transferring to society the results of their research, since there are indeed many problems that can benefit from a better understanding of language facts, from clinical linguistics, to artificial intelligence, to cultural mediation.

Overall, because the field is increasingly multidisciplinary, methodologically complex, theoretically diverse, and applicable, and because research in all areas is growing exponentially, it is difficult for researchers to be up to date. Accordingly, reliable, open-source summaries of topics of interest for the field are more needed and more welcome than ever. The aim of this Research Topic is to gather comprehensive and actualized reviews of issues of particular relevance for language sciences. We have brought together 6 contributions from 14 scholars.

Reflecting current deep interest in second language learning, we have four reviews of specific areas of research on second language learning: Wang on memorization strategies, and, in particular, on the recent move to memorization of longer texts, rather than simply individual words; Dou et al. on approaches to teaching second languages, particularly on the recent switch to English for Specific Purposes; Klimova and Seraj on the current role and future promise of Chatbots in teaching second languages; and Qiao on the factors that affect second language learners, including motivation, aptitude, personality, intelligence, and learner preferences.

In addition, Maggu et al. present a meta-analytic review of work on the role of complex input in children with speech sound disorders, reflecting the importance of language science in providing clinical insights and treatments.

Finally, Wie and Knoeferle review literature on how language relates to events in the world, their causal connections, and their representations, reflecting the deeply related nature of language and cognition.

Given our interest in providing good, open-source, scientifically valuable reviews of the most current areas of language research, broadly conceived, we wish to end by drawing attention to another Frontiers in Psychology Research Topic: “*Reviews in psychology of language*,” which we hope will continue to provide a valuable resource to researchers in the area looking for comprehensive reviews across all areas of research in the psychology of language.

## Author contributions

AB-B: Writing – original draft, Writing – review & editing. AB: Writing – original draft, Writing – review & editing. TS: Writing – original draft, Writing – review & editing.

